# Heterologous expression of naturally evolved putative *de novo* proteins with chaperones

**DOI:** 10.1002/pro.4371

**Published:** 2022-07-13

**Authors:** Lars A. Eicholt, Margaux Aubel, Katrin Berk, Erich Bornberg‐Bauer, Andreas Lange

**Affiliations:** ^1^ Institute for Evolution and Biodiversity University of Muenster Münster Germany; ^2^ Max Planck‐Institute for Biology Tuebingen Tübingen Germany

**Keywords:** chaperones, *de novo* protein, disorder and secondary structure prediction, *Drosophila melanogaster*, *Homo sapiens*, Western blot

## Abstract

Over the past decade, evidence has accumulated that new protein‐coding genes can emerge *de novo* from previously non‐coding DNA. Most studies have focused on large scale computational predictions of *de novo* protein‐coding genes across a wide range of organisms. In contrast, experimental data concerning the folding and function of *de novo* proteins are scarce. This might be due to difficulties in handling *de novo* proteins *in vitro*, as most are short and predicted to be disordered. Here, we propose a guideline for the effective expression of eukaryotic *de novo* proteins in *Escherichia coli*. We used 11 sequences from *Drosophila melanogaster* and 10 from *Homo sapiens*, that are predicted *de novo* proteins from former studies, for heterologous expression. The candidate *de novo* proteins have varying secondary structure and disorder content. Using multiple combinations of purification tags, *E. coli* expression strains, and chaperone systems, we were able to increase the number of solubly expressed putative *de novo* proteins from 30% to 62%. Our findings indicate that the best combination for expressing putative *de novo* proteins in *E. coli* is a GST‐tag with T7 Express cells and co‐expressed chaperones. We found that, overall, proteins with higher predicted disorder were easier to express.

**Statement:**

Today, we know that proteins do not only evolve by duplication and divergence of existing proteins but also arise from previously non‐coding DNA. These proteins are called *de novo* proteins. Their properties are still poorly understood and their experimental analysis faces major obstacles. Here, we aim to present a starting point for soluble expression of *de novo* proteins with the help of chaperones and thereby enable further characterization.

## 1 INTRODUCTION

*De novo* genes originate from intergenic or non‐coding DNA regions^1^, ^2^, ^3^, ^4^, ^5^, ^6^, ^7^ in contrast to genes that emerge by duplication^8^, ^9^ or rearrangement from existing gene fragments.^10^ Therefore, recent, true *de novo* genes have no precursor by definition and have not been subjected to selection for particular structures or functions for long, if at all. Due to their recent emergence, *de novo* genes tend to be shorter, evolve more rapidly, and have lower expression than established genes.^3^, ^4^ Short‐length and accelerated evolution make it difficult to reliably detect (or reject) homologs of orphan genes and thereby identify true *de novo* genes. By combining homology and synteny based approaches for *de novo* gene identification, the origin of *de novo* genes can be detected more accurately.^11^

Several *de novo* protein‐coding genes have been identified and confirmed across a wide range of eukaryotes.^12^, ^13^, ^14^, ^15^, ^16^, ^17^, ^18^, ^19^, ^20^, ^21^, ^22^ These *de novo* genes were mainly analyzed with comparative genomics and transcriptomics. A recent study by Grandchamp et al.^23^ showed that proto‐genes, an intermediate step in *de novo* gene emergence,^24^ contain regulatory sequences similar to established genes. Depending on the genomic position of the recently emerged proto‐gene, introns may already be present in the proto‐gene, making it harder to distinguish from established genes. However, without experimental evidence on structure and function, our evolutionary understanding of how *de novo* proteins emerge, remains incomplete.

Difficulties in handling *de novo* proteins, together with the novelty of the research area, might be the reason for the lack of experimental studies on *de novo* proteins. So far only two *de novo* proteins were expressed and characterized experimentally, Goddard (Gdrd)^25^ and Bsc4.^26^ In both cases, the expressed *de novo* protein was difficult to analyze due to unstable or incorrect folding (Bsc4) or unusual behavior in SDS‐PAGE (Gdrd). Compared to well‐studied proteins with expression and purification data available, *de novo* proteins tend to behave differently when using standard protocols.

Several studies, foremost some from the laboratory of Dan Tawfik,^27^, ^28^, ^29^, ^30^ inspired us to apply co‐expression with chaperones to achieve soluble expression of *de novo* proteins. Since *de novo* proteins evolve rapidly by becoming coding from scratch, they probably lack a stable structural configuration and contain high amounts of disorder.^3^, ^4^ Those properties determine the levels of soluble and insoluble fractions of a protein during *in vitro* experiments and could explain the obstacles faced during their expression.^31^, ^32^ On the other hand, it is not yet clear if *de novo* proteins undergo a similar hindrance in their native organism or only in the expression hosts.^33^ While Tawfik and colleagues used chaperones to explore the sequence space of enzymes and enable soluble expression of mutants,^27^, ^28^, ^29^ we hypothesized that *de novo* protein expression might also profit from chaperones. With their “emergence from dark genomic matter” in the DNA^34^ and predicted lack of stability and high disorder, *de novo* proteins are prospective targets for chaperones because their solubility can be increased.^27^, ^28^ Increased solublity can be relevant for protein purification and any follow‐up experiments.

The chaperonin GroEL and its co‐chaperone GroES are found throughout the bacterial domain, while their homologs, HSP60 and HSP10, respectively, are found in eukaryotes.^35^ GroEL/GroES play a pivotal role in the translocation, dis‐aggregation, function, and folding of newly synthesized peptides after translation.^27^, ^35^, ^36^, ^37^

**FIGURE 1:**
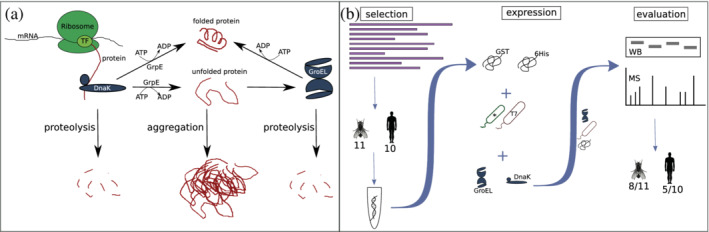
(a) Mechanism of chaperone assisted protein folding after Thomas et al.^38^ The nascent protein is bound by the DnaK/J complex and release is triggered by GrpE under ATP hydrolysis. After release, the protein is either correctly folded, degraded (proteolysis), or remains unfolded. The unfolded protein can either aggregate or bind to the GroEL/ES complex. GroEL/ES either releases the folded protein by ATP hydrolysis or the protein is degraded. (b) Overview of the workflow on *de novo* protein expression: We first selected candidate proteins from *Drosophila melanogaster* (11, including Atlas) and 10 from *Homo sapiens* from a pool of putative *de novo* genes for expression. The 21 sequences were codon optimized for *E. coli* and ordered from Twist. For expression, different tags (GST and His), different *E. coli* expression cells (star, T7), and different chaperones (GroEL and DnaK systems) were tested. The success of protein expression was verified by Western blot (WB) and mass spectrometry (MS)

The other chaperone system used here is DnaK, DnaJ, and GrpE (homologous to HSP70 and HSP40 in eukaryotes). For simplicity we will refer to the chaperone system GroEL/ GroES as only GroEL and to DnaK, DnaJ, and GrpE as DnaK only. While the GroEL system targets misfolded and unfolded proteins, DnaK can refold an already aggregated protein to its native state using ATP (see Figure 1a).^39^, ^40^, ^41^, ^42^ The two different chaperone systems can be exploited for challenging heterologous expression of proteins which are foreign to the host and thus prevent misfolding and aggregation which is often associated with heterologous expression.^27^, ^28^, ^29^, ^35^, ^41^, ^43^

For this study, we used 21 putative *de novo* proteins, 11 from *Drosophila melanogaster* (termed here as *DM*1‐10 and Atlas) and 10 from *Homo sapiens* (termed here as *HS*1‐10) as shown in Figure 1b). The sequences of all used putative *de novo* proteins can be found on Zenodo (https://doi.org/10.5281/zenodo.6512224), for genomic location and official gene names see Table S1. These *de novo* proteins have been recently published by Heames et al.^21^ and Dowling et al., respectively.^22^ Additionally, we tested our method on a recently published and better characterized putative *de novo* protein from *D. melanogaster*, called Atlas. Atlas appears to function as a DNA binding protein that facilitates the packaging of chromatin in developing *D. melanogaster* sperm.^44^ Since experimental work with *de novo* proteins is still underrepresented (compared with computational studies) and challenging, we want to propose a guideline for successful expression of putative *de novo* proteins in *E. coli*. We combined different chaperone systems (GroEL and DnaK) with different combinations of *E. coli* strains (BL21 Star™ [DE3] and T7 Express) in order to express putative de novo proteins solubly. To verify successful expression of target proteins, Western blots were performed and samples sent for tryptic digest followed by mass spectrometry. We identified the best combination for expression of putative *de novo* proteins in *E. coli*. After first expressions with His‐tag alone resulted in soluble expression for only 1/21 proteins, we increased the total number of solubly expressed putative *de novo* proteins to 13/21 with GST‐tag and chaperones. The different chaperone systems increased or enabled soluble expression in four cases, while DnaK only helped in two, GroEL in all of those four.

## 2 RESULTS

### 2.1 Structural content of the putative *de novo* proteins

#### 2.1.1 Disorder predictions

**FIGURE 2:**
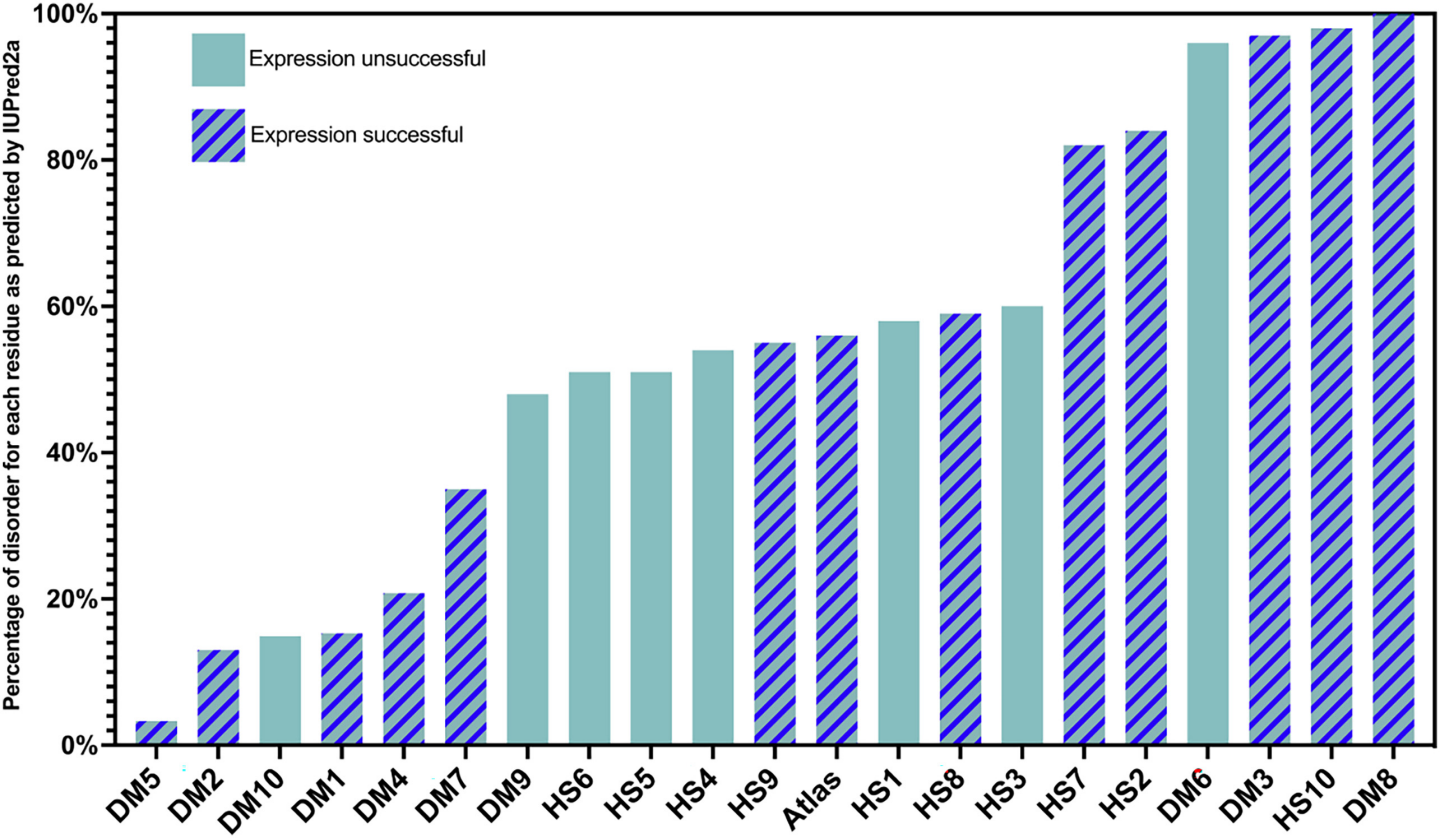
Percentage of disorder as calculated with IUPred2a. All candidate *de novo* proteins used for expression experiments ordered by their disorder level from the left to right. Unicolor bars belong to the unsuccessfully expressed proteins, striped bars to the successfully expressed ones

**FIGURE 3:**
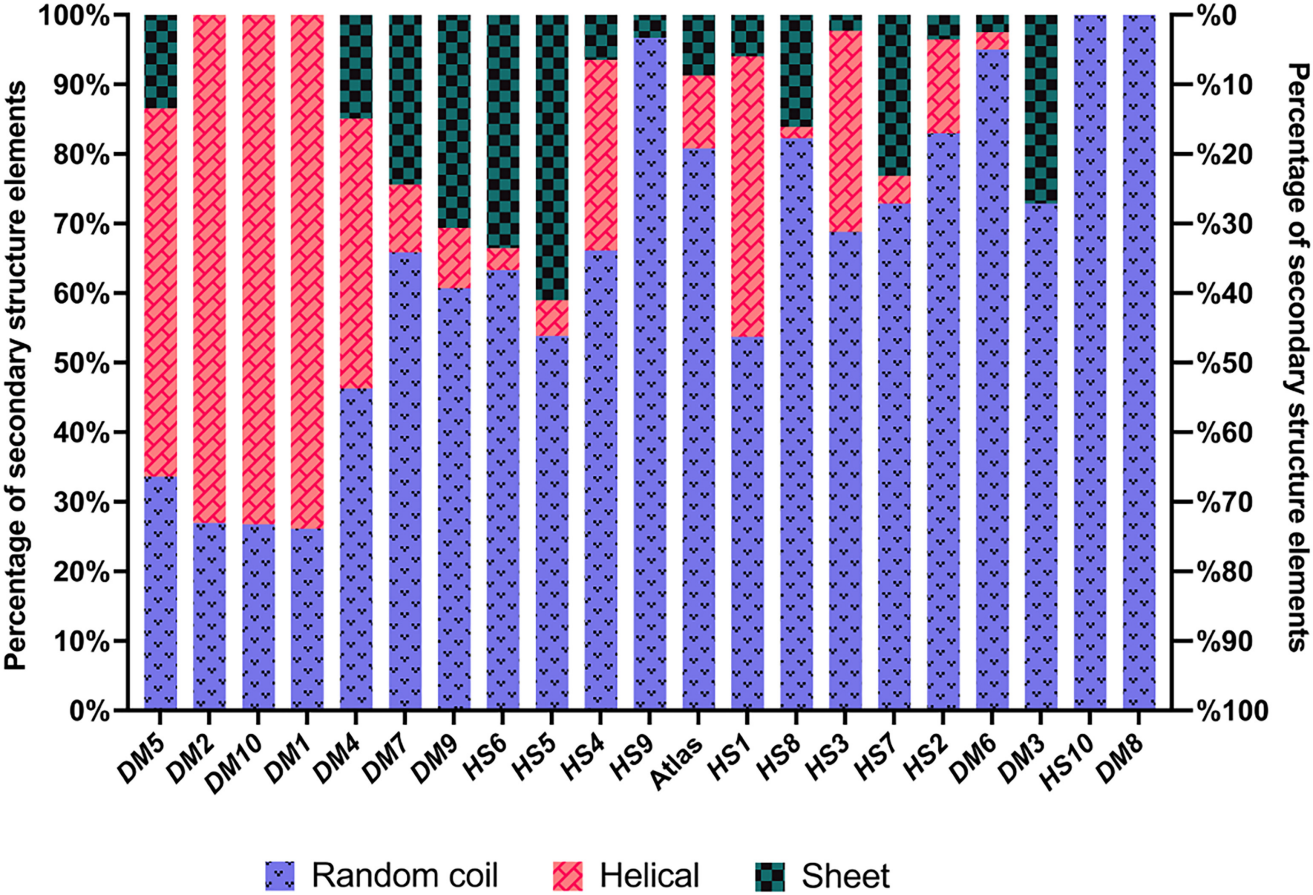
Percentage of random coils, α‐helices, and β‐sheets predicted by Porter 5.0 for each *de novo* protein candidate. Left to right following increasing disorder level based on Figure 2

We performed disorder predictions with IUPred2a^45^, ^46^ on all candidate *de novo* proteins. For this we calculated the percentage of residues predicted to be disordered (Figure 2), as opposed to the overall average disorder score (Figure S1). This allows direct comparison to secondary structure predictions (Figure 3). Our first objective here was to choose candidate *de novo* proteins with different levels of intrinsic disorder to observe any difference in their ability to express. If any trend in predicted disorder and soluble expression or susceptibility to chaperones was observed, this could help choosing promising candidates for characterization in future experiments. The predicted disorder ranged from around 3%–100% as shown in Figure 2. *DM*5 was predicted to have least disorder content, while *DM*6, *DM*3, *HS*10, and *DM*8 appear to be entirely disordered. The putative *de novo* protein Atlas has predicted disorder of 60%.

#### 2.1.2 Secondary structure predictions

Predictions of secondary structure elements were performed using Porter 5.0^47^, ^48^ for all candidate proteins and are shown in Figure 3. The predicted random coils should be equivalent to the disordered regions predicted by IUPred2a.^49^ While the results indicate a high amount of random coils for most candidates, they do not completely follow the trend of the disorder predictions by IUPred2a (compare Figure 2). *DM*3, for example, is predicted to be ∼100% disordered by IUPred2a, while, on the other hand, it is predicted to have over 20% β‐sheet and ∼70% random coils by Porter 5.0.

Our goal was to choose a cohort of *de novo* proteins that consist of a diverse range in composition of structural elements. We assumed that a protein containing more secondary structure elements should be better accessible for soluble expression with chaperones.^50^ Notably, *DM*1 (∼70% α‐helical), *DM*2 (∼70% α‐helical), *DM*4 (∼55% α‐helical, ∼10% β‐sheets), *DM*5 (∼60% α‐helical, ∼10% β‐sheets), and *DM*10 (∼70% α‐helical, ∼10% β‐sheets) are predicted to have secondary structure contents of 50% or more, with α‐helices to be more frequent than β‐sheets and less than 50% random coils. *HS*1 (∼50% random coils, ∼45% α‐helical, ∼5% β‐sheets), *HS*3 (∼70% random coils, ∼25% α‐helical, ∼5% β‐sheets), *HS*4 (∼65% random coils, ∼30% α‐helical, ∼5% β‐sheets), *HS*5 (∼55% random coils, ∼5% α‐helical, ∼40% β‐sheets), *HS*6 (∼60% random coils, ∼5% α‐helical, ∼35% β‐sheets), *HS*7 (∼70% random coils, ∼5% α‐helical, ∼25% β‐sheets), *DM*3 (∼70% random coils, ∼30% sheets), *DM*7 (∼65% random coils, ∼10% α‐helical, ∼25% β‐sheets), and *DM*9 (∼60% random coils, ∼10% α‐helical, ∼30% β‐sheets), on the other hand, are predicted to be mostly random coils (disordered) with otherwise higher amounts of β‐sheets predicted. *DM*6 (∼90% random coils), *DM*8 (∼100% random coils), *HS*2 (∼85% random coils), *HS*9 (∼95% random coils), and *HS*10 (∼100% random coils) are predicted to contain more or less only random coils.

### 2.2 Expression of putative *de novo* proteins

#### 2.2.1 Candidate proteins of *Drosophila melanogaster*

Our initial approach was similar to the successful expression of characterized putative *de novo* protein Gdrd.^25^ Therefore, we aimed to express our **11** putative *de novo* protein candidates with an N‐terminal 6xHis‐tag in *E. coli* BL21 Star™ (DE3) cells, and verify expression via SDS‐PAGE and mass spectrometry. However, for our candidates, the expression level was either very low or not detectable, as can be seen in Figure S3. We switched to different *E. coli* cells (T7 Express), but expression remained unsuccessful. Shifting from an N‐terminal 6xHis‐Tag to a C‐terminal 6xHis‐tag showed similar negative results. Considering the size and levels of disorder, we switched to a larger tag for increased solubility and stability, choosing an N‐terminal GST‐tag. In this way, we were able to observe a higher success rate in soluble expression of our target proteins. But not all proteins could be expressed at satisfying levels, especially solubility needed to be increased for some (Figure S3).

**FIGURE 4:**
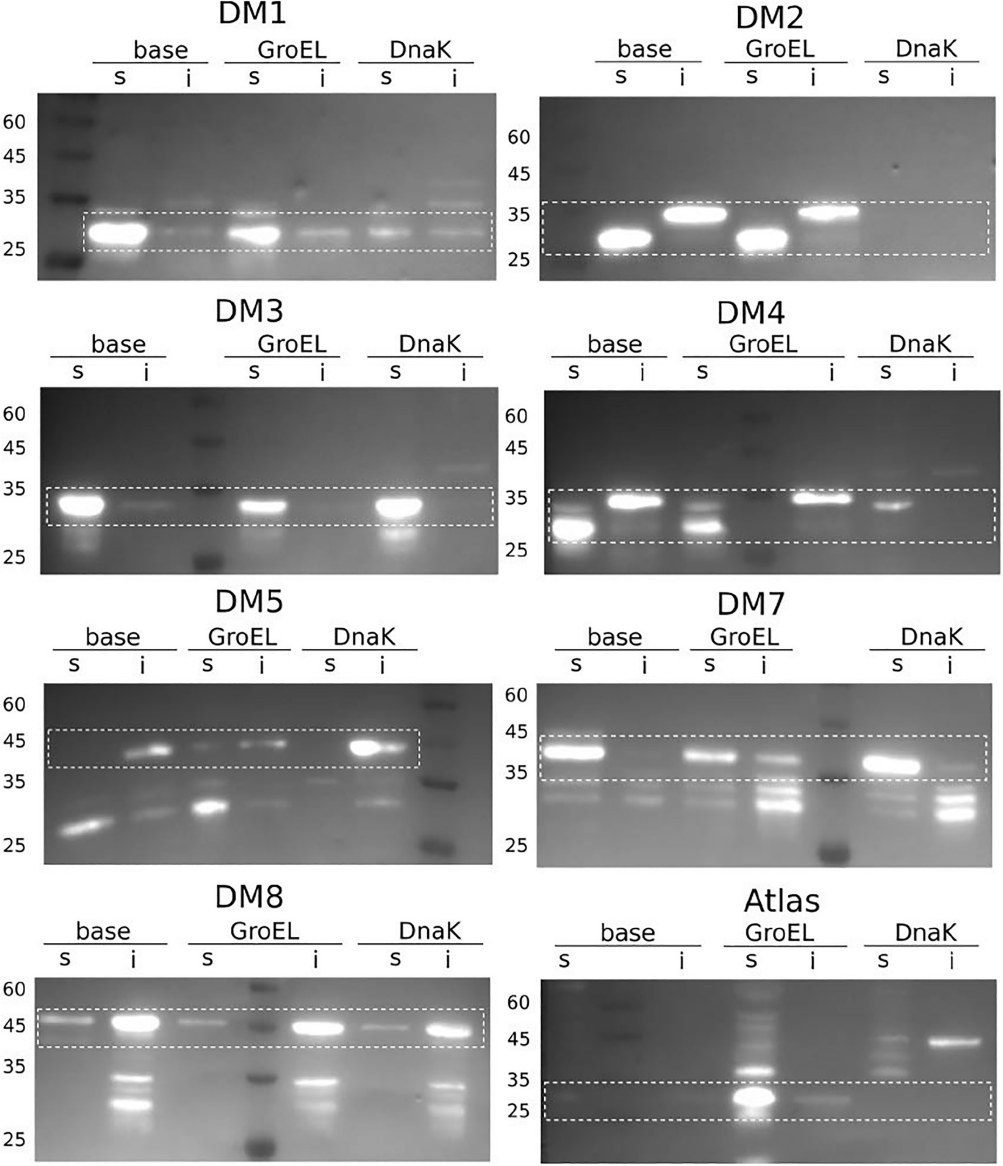
Western blots with anti‐His antibody. Boxes indicate the height of the target protein band: ***DM*1** (34 kDa): highest solubilty without chaperones, then GroEL, then DnaK; highly soluble. ***DM*2** (36 kDa): only insoluble, even with chaperones. ***DM*3** (33 kDa): DnaK highest solubilty, then base, then GroEL; very soluble. ***DM*4** (34 kDa): DnaK highest solubilty, then GroEl, then base; very insoluble. ***DM*5** (39 kDa): GroEL only one with soluble fraction, runs a bit high. ***DM*7** (36 kDa): Dnak highest solubilty, then base, then GroEL very soluble. ***DM*8** (37 kDa): all similar, different expression levels, first base, then GroEL, then DnaK; more insoluble. **Atlas** (20 kDa): GroEL highest solubilty, nothing in base and DnaK

Inspired by successful work carried out by Tawfik et al.,^27^, ^28^, ^29^ we hypothesized that chaperones could improve thermodynamic stability of these evolutionarily young proteins thus enabling their soluble expression. We repeated our experiments with the addition of the two chaperone systems (i) GroEL and (ii) DnaK. We were able to increase the number of solubly expressed *de novo* candidate proteins of *D. melanogaster* using the combination of either GroEL or DnaK and N‐terminal GST‐tag (see Figure 4). However, for the candidate proteins *DM*6, *DM*9, and *DM*10 no soluble expression was achievable, despite the use of different tags, strains, or chaperones. Only in the case of Atlas, the combination of N‐terminal 6xHis‐tag and GroEL worked best. We tested all combinations in BL21 Star™ (DE3) and T7 Express *E. coli* cells. Six candidate proteins were expressed in T7, two were expressed in BL21 Star™ (DE3) cells. Three proteins were not expressable in either strain. In summary, with the combination of chaperones and switching to N‐terminal GST‐tag, we were able to express 8/11 of the *D. melanogaster* putative *de novo* protein candidates (see Table 1).

**TABLE 1:** Expression conditions and results of *D. melanogaster de novo* proteins. Base = no chaperones, GroEL = GroEL/ES, DnaK = DnaK/J/GrpE. Plus signs mean visible expression, two plus signs strong expression, 0 means no visible expression. Arrows indicate the change in expression with added chaperones in comparison with base

Protein	Cell/tag	Base	GroEL	DnaK	GroEL effect	DnaK effect	Disorder (%)
*DM*1	T7/GST	+ +	+ +	+	−	*↓*	15
*DM*2	Star/GST	+ +	+ +	0	−	*↓*	13
*DM*3	T7/GST	+ +	+	+ +	*↓*	−	97
*DM*4	T7/GST	+ +	+	+	*↓*	*↓*	21
*DM*5	T7/GST	0	+	0	*↑*	−	3
*DM*6	−/−	0	0	0	−	−	97
*DM*7	T7/GST	+ +	+	+ +	*↓*	−	35
*DM*8	T7/GST	+	+	+	−	−	100
*DM*9	−/−	0	0	0	−	−	49
*DM*10	−/−	0	0	0	−	−	15
Atlas	Star/6xHis	0	+ +	0	*↑*	−	56

#### 2.2.2 Comparison of different chaperone conditions for *D. melanogaster* proteins

Western blots were used for comparison of the soluble expression levels with and without chaperones, in order to test our hypothesis that chaperones would increase soluble expression of the target proteins. The optimal conditions identified by SDS‐PAGEs were repeated under three settings: (i) without chaperones (base), (ii) with GroEL, and (iii) with DnaK. Surprisingly, we did not observe increased solubility for most putative *de novo* proteins when adding chaperones (see Figure 4 and Table 1).

In contrast, we observed soluble expression for most proteins without chaperones, for example, *DM*1, *DM*2, *DM*3, *DM*4, and *DM*7. In combination with GroEL, the intensity of the bands in the soluble fraction and therefore amount of soluble protein, even decreased for *DM*3, *DM*4, and *DM*7. For *DM*2 and *DM*5, the amount of soluble protein increased when co‐expressed with GroEL. When DnaK was co‐expressed, protein solubility either appeared to decrease (*DM*1, *DM*2, and *DM*4) or was similar to the base (*DM*3 and *DM*7). *DM*8 showed similar soluble expression for all three conditions with most of the protein being insoluble. In the case of Atlas and *DM*5, soluble protein expression was increased or enabled with the addition of the GroEL chaperone system while DnaK and base expression resulted in no or very little soluble protein. While we cannot confirm that co‐expression with DnaK in fact decreases the amount of soluble protein (*DM*1, *DM*2, and *DM*4), we do not see increased soluble expression for any of the candidate proteins in the presence of DnaK as we do for GroEL (*DM*5 and Atlas).

#### 2.2.3 Candidate proteins of *Homo sapiens*

The 10 putative human *de novo* proteins were expressed following the same protocol as the *D. melanogaster* proteins by combining the different *E. coli* expression cells, tags, and chaperone systems (Figure S4). We detected a similar trend here as for the *D. melanogaster* proteins (N‐terminal GST‐tag in *E. coli* T7 express cells; see Table 2). One protein (*HS*8), however, was only weakly expressed with an N‐terminal 6xHis‐tag but using also *E. coli* T7 express cells. Without the addition of chaperones only *HS*7, *HS*8, and *HS*10 were successfully expressed and soluble. After co‐expression with chaperones, as described for *D. melanogaster* proteins, two more *H. sapiens* proteins could be expressed. Unfortunately, *H. sapiens* protein candidates *HS*1, *HS*3, *HS*4, *HS*5, and *HS*6 showed no expression at all, even with chaperones. In total, we were able to express 5 out of 10 putative *de novo* proteins following our protocol (see Table 2).

**TABLE 2:** Expression conditions and results of *H. sapiens de novo* proteins. Base = no chaperones, GroEL = GroEL/ES, DnaK = DnaK/J/GrpE. Plus signs mean visible expression, two plus signs strong expression, 0 means no visible expression. Arrows indicate the change in expression with added chaperones in comparison with base

Protein	Cell/tag	Base	GroEL	DnaK	GroEL effect	DnaK effect	Disorder (%)
*HS*1	−/−	0	0	0	−	−	59
*HS*2	T7/GST	0	+	+	*↑*	*↑*	84
*HS*3	−/−	0	0	0	−	−	60
*HS*4	−/−	0	0	0	−	−	54
*HS*5	−/−	0	0	0	−	−	51
*HS*6	−/−	0	0	0	−	−	51
*HS*7	T7/GST	+ +	+ +	0	−	*↓*	82
*HS*8	T7/6xHis	+	+	+	−	−	60
*HS*9	T7/GST	0	+	+	*↑*	*↑*	56
*HS*10	T7/GST	+	+	+	−	−	99

#### 2.2.4 Comparison of different chaperone conditions for *H. sapiens* proteins

**FIGURE 5:**
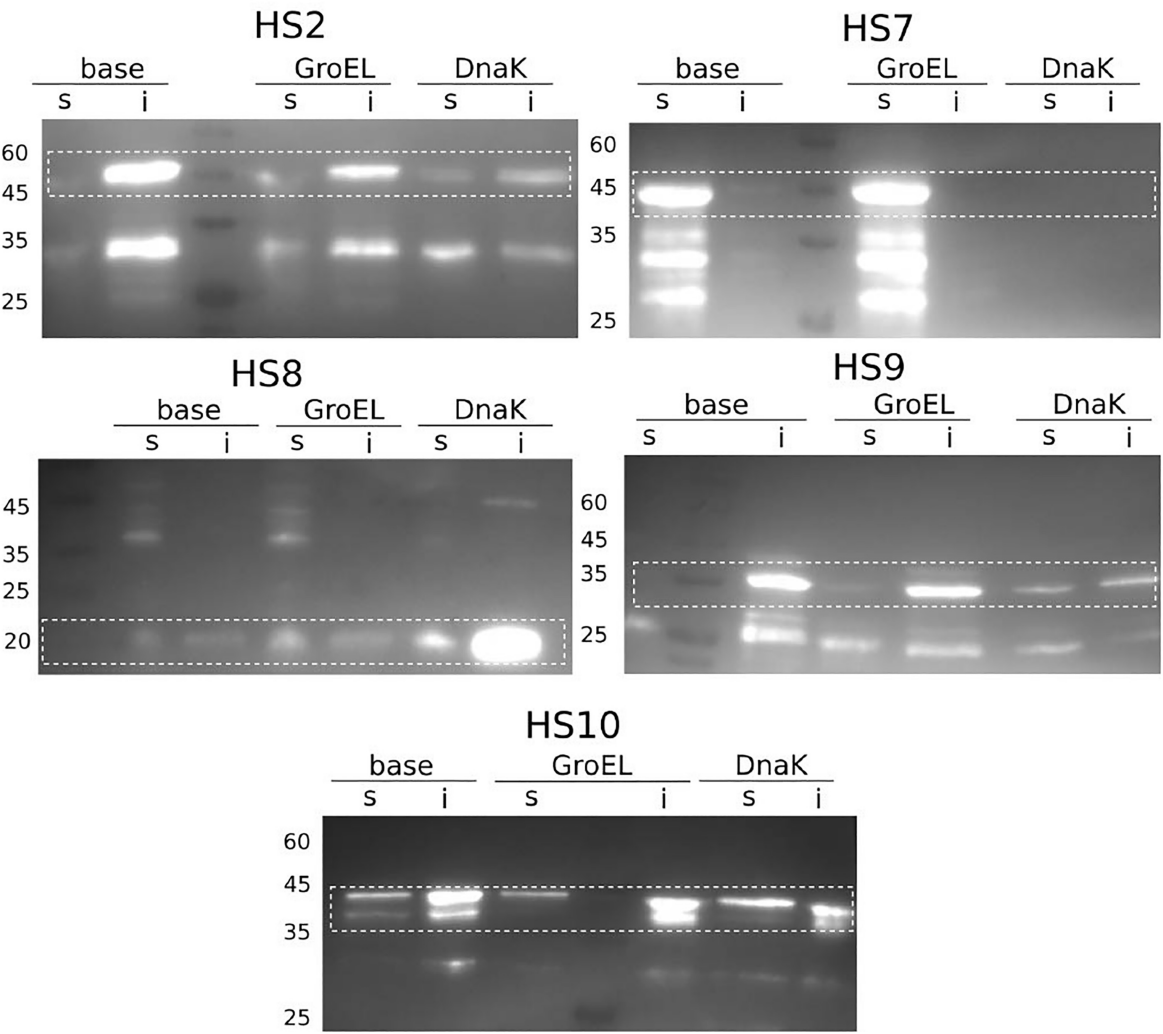
Western blots with anti‐His antibody. Boxes indicate the height of the target protein band: ***HS*2** (44 kDa): upper bands (lower are degraded protein or double bands) most in DnaK, then GroEL, then base; very insoluble. ***HS*7** (50 kDa): GroEL best, then base, nothing in DnaK. Possible protein degradation; very soluble. ***HS*8** (16 kDa): upper bands most in DnaK, then GroEL, then base; very insoluble. ***HS*9** (42 kDa): upper bands (lower are degraded protein or double bands) most in DnaK, then GroEL, then base; very insoluble. ***HS*10** (43 kDa): upper bands (lower are degraded protein or double bands) most in GroEL, then DnaK, then base; very insoluble

Western blots were used for comparison of the three different chaperone expressions (i) base, (ii) GroEL, and (iii) DnaK, as described above. Two out of the five successful candidates (*HS*2 and *HS*9) showed very weak or no soluble expression without chaperones, but solubility could be increased with both chaperone systems. *HS*8 and *HS*10 showed low soluble expression overall, but no change in solubility was visible when co‐expressing with either chaperone system. The candidate *de novo* protein *HS*7 already showed strong soluble expression at base (Figure 5). However, the addition of GroEL seemed to increase soluble expression further, while DnaK co‐expression led to low or no protein being detected. Overall, the trend observed for the *D. melanogaster* proteinś was consistent with the trend observed for the *H. sapiens* proteins. GroEL increased soluble expression for most putative *de novo* proteins while DnaK lacked substantial influence on protein solubility.

## 3 DISCUSSION

*De novo* proteins have first been detected more than a decade ago and the mechanism of their emergence has been studied intensely ever since.^4^, ^12^ Still, there are concerns (i) regarding the reliability of their computational identification^24^, ^51^, ^52^ and (ii) if and how they code for functional proteins. To shed light on these concerns, *de novo* proteins need to be studied experimentally and theoretically. The handling of *de novo* proteins by heterologous expression and purification is often difficult because solubility is low and purification yields little amounts and potentially unstable proteins. Moreover, identifying the function of these young genes, is another challenging task. In this study, we present a guideline for expressing *de novo* proteins in *E. coli*.

### 3.1 Expression cells

*E. coli* is the most widely used model organism for recombinant expression. However, foreign proteins can be toxic to *E. coli* by interfering with the physiology or leading to protein aggregation. This may result in low expression yields, growth defects, or even cell death.^53^, ^54^, ^55^ Optimized expression hosts and plasmids^53^, ^54^, ^55^ or chaperones can be used to overcome the expression issues caused by proteins which are a metabolic burden for the host. Here, we used two different types of the *E. coli* strains (DE3): BL21 Star™ and T7 Express. Both strains resulted in effective protein expression and a relatively high yield of the *de novo* proteins, with T7 Express being the best option. The de novo proteins studied here are possibly a toxic, metabolic burden to the *E. coli* cells, suggesting T7 cells are the better choice of expression cell. BL21 Star™ (DE3) contains a T7‐RNA‐polymerase under control of lacUV5 promoter together with higher mRNA stability. This leads to stable mRNA transcripts and higher amount of target protein. However, BL21 Star™ (DE3) cells have increased basal expression of heterologous genes and cannot express toxic genes. In contrast, the T7 Express cells have a reduced basal expression of target proteins than BL21 Star™ (DE3) cells. Therefore, toxic proteins can be expressed better in T7 cells compared with BL21 Star™ (New England Biolabs).^56^

### 3.2 Comparing different protein tags

Based on our study, an N‐terminal GST‐tag was the more appropriate choice than a 6xHis‐tag. Some *de novo* protein candidates are quite small (8–12 kDa), so a larger tag like GST might already stabilize in a chaperone‐like manner.^55^, ^57^ However, Atlas and *HS*8, that is, 2/21, were only expressed with an N‐terminal 6xHis‐tag. With a mass of only 1 kDa, 6xHis‐tag is the better choice for further structural characterization using circular dichroism (CD), multi‐angle light scattering (MALS) or nuclear magnetic resonance (NMR), since a small tag has less influence on protein folding. In contrast, the larger GST‐tag needs to be cleaved for most follow‐up experiments. When removing the tag, the *de novo* protein might behave differently and could degrade or aggregate.

### 3.3 Influence of chaperones on protein expression and solubility

**FIGURE 6:**
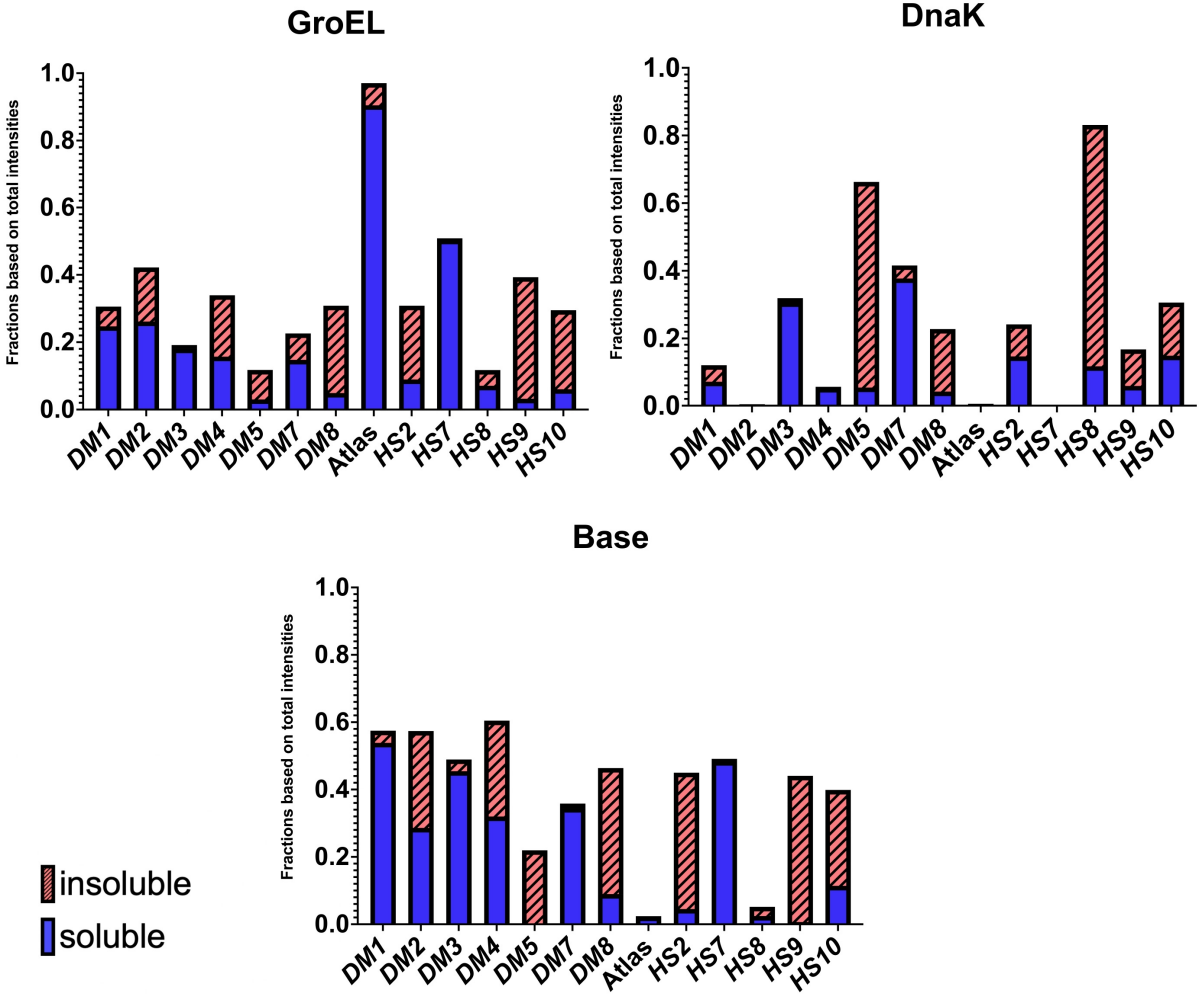
Fractions of soluble and insoluble expression for candidate *de novo* proteins with expression of GroEL or DnaK and without (base). Values are intensities of Western blot bands

Our Western blot results indicate that GroEL slightly outperforms DnaK in terms of increased protein solubility. In some cases, both chaperone systems increase or enable soluble expression (*HS*2 and *HS*9, 2/21) but for most proteins GroEL leads to more soluble protein than DnaK (*DM*1, *DM*2, *DM*5, Atlas, and *HS*7, 5/21) (Figure 6). DnaK requires easily accessible hydrophobic fragments that can be predicted from the protein sequence, while GroEL demands no defined binding motifs. However, in the case of our proteins, we found no connection between predicted DnaK binding sites and influence of DnaK on protein expression level (Figure S2). While GroEL could increase solubility for single *de novo* proteins in 5/21 cases, this system was not successful in Heames et al.,^7^ in which we used a library of 1800 putative *de novo* proteins (4–8 kDa) in a cell‐free expression system.

In some cases (*DM*1, *DM2*, *HS*7, and Atlas), DnaK decreased the solubility of proteins that expressed soluble without chaperones. One could assume that DnaK and target protein expression compete for cellular ressources and that for already soluble proteins this has no positive trade‐off. Another reason could be DnaK's role as central organizer in the chaperone network of *E. coli*. Binding to DnaK could trigger degradation of the “toxic” target protein but also overexpression of DnaK additionally to the endogenous version could bring the cellular metabolism out of balance.^58^, ^59^

We cannot verify that changes with co‐expression of chaperones is solely due to effects of chaperones on putative *de novo* proteins or on overall amount of protein expression. Our main interest here is to optimize expression for follow‐up experiments and not to draw general conclusions on chaperone interaction with *de novo* proteins.

Drawing conclusions from heterologous expression experiments toward *in vivo* interactions of proteins and chaperone systems are fragmentary and can only serve as hypotheses in need of further verification using *in vivo* experiments.^60^

### 3.4 Comparing putative de novo proteins from *D. melanogaster* to *H. sapiens*

In total, we were able to successfully express 13 out of 21 putative *de novo* proteins in *E. coli* cells (eight in *D. melanogaster* and five in *H. sapiens*). For both, *D. melanogaster* and *H. sapiens* candidate putative *de novo* proteins, the combination of GST‐tag and *E. coli* T7 Express cells were the best performing (10 out of 13). We performed test expressions and compared the levels of soluble expression for different chaperone combinations shown in Figures 4 and 5. Expression results from putative *de novo* protein candidates *DM*5, Atlas, *HS*2, and *HS*9 were in line with our original hypothesis that chaperones enhance solubility of *de novo* proteins in heterologous expression systems. However, the choice of appropriate tag and expression cells in the first step was equally, if not more, important. When using the N‐terminal His‐tag that proved successful for putative *de novo* protein Gdrd, only two (Atlas and *HS*7) of our candidate proteins were expressed. When switching to the N‐terminal GST‐tag another seven *D. melanogaster* and four more *H. sapiens* protein candidates were expressed. Unfortunately, we were not able to express 8/21 of the candidate proteins in *E. coli* at all (*HS*1, *HS*3 – *HS*6, *DM*6, *DM*9, and *DM*10), despite trying different expression strains, tags and chaperone systems.

### 3.5 Disorder and secondary structure predictions

When examining the predicted structural properties of the human *de novo* protein candidates, we observe a slight trend toward better expression of the more disordered proteins. This trend can be observed for the IUPred2a disorder predictions (Figure 2) but becomes more apparent for the overall secondary structure predictions (Figure 3). The unsuccessful expression candidates *HS*1, *HS*3, and *HS*4 showed a higher predicted α‐helical content of approximately 40% while *HS*5 and *HS*6 had a higher predicted β‐sheet content of around 30%–40% compared with the other human candidate proteins *HS*2, *HS*7, *HS*8, *HS*9, and *HS*10) which are predicted to contain over 70% random coils (or 60% disorder). The described differences in predicted secondary structure content and disorder level might be the reason why these putative *de novo* candidates could not be expressed in *E. coli* cells even with the help of chaperones.

For *D. melanogaster* protein candidates, this trend was not observed. Here, several of the proteins with lower disorder predicted (*DM*1, *DM*4, and *DM*7) were expressed solubly without addition of chaperones. Yet, *DM*6 (∼90% disorder predicted) was not expressed successfully. However, the two proteins with 100% random coils predicted by Porter 5.0 and highest disorder predictions by IUPred2a (*DM*8 and *HS*10) did not show any change in solubility when chaperones were co‐expressed. Considering that such highly disordered proteins do not need chaperones, this observation was expected.

Deviations of the level of predicted disorder and predicted secondary structures, especially random coils, for each protein can be explained by the differences in IUPred2a and Porter 5.0. IUPred2a provides energy estimations for each amino acid residue resulting in quasi‐probabilities of disorder.^46^ On the other hand, Porter 5.0 is based on a neural network relying on sequence alignments and co‐evolutionary information.^47^ These fundamentally different approaches can lead to inconsistent results in some cases (e.g., *HS*9 and *DM*3) while not invalidating one another.

## 4 CONCLUSION AND OUTLOOK

Exemplifying the general trend for soluble *de novo* protein expression is only the first step toward enabling further *in vitro* experiments for functional and structural characterization. Further advancement will lead to efficient and stable purification, followed by functional assays such as peptide phage display to identify binding partners.^61^, ^62^ This technique has proven to be useful for high‐throughput screening of intrinsically disordered regions for short linear motifs,^63^ especially for human proteins. Soluble expression and purification will be crucial for structural characterization via CD, NMR, and Cryo‐EM. Due to their small size and high disorder content, only NMR^25^ and potentially Cryo‐EM^64^ will be capable of solving the structure of *de novo* proteins experimentally. Even in light of the recent dawn of computational structure prediction,^65^, ^66^ experimental structural and functional determination remains necessary, especially for *de novo* proteins. While contemporary prediction methods can certainly provide a first estimate on structure, the intrinsic nature of *de novo* proteins, with their short length, high disorder content and lack of homology, will demand some scepticism while analyzing such predictions.^67^, ^68^ Surprisingly, the AlphaFold prediction of Goddard (GEO12017p1, AlphaFold Protein Structure Database)^69^ is in line with its partial experimental characterization^25^ and its central helix is predicted with high confidence. Despite the lack of homology, which is a core demand for the MSA generation of AlphaFold2 and a hallmark of *de novo* proteins, one could assume that *de novo* proteins are of such small size that AlphaFold can solve their local folding. This will have to be validated in future studies. This study of 21 putative *de novo* proteins from *H. sapiens* and *D. melanogaster*, including previously *in vivo* characterized putative *de novo* protein Atlas, showed that chaperones may help expressing *de novo* proteins in *E. coli* cells. However, not all putative *de novo* proteins needed chaperones for soluble expression and sometimes even expressed better without. Fusion of the target *de novo* proteins to a GST‐tag and using T7 Express cells as hosts proved to be the most successful combination. Our work may serve as a guide to facilitating future analyses of putative *de novo* proteins or other difficult (short and/or disordered) target proteins in *E. coli*.

## 5 MATERIALS AND METHODS

### 5.1 Online data availability

All SDS‐PAGEs, MS results, Western blots, and scripts are deposited in Zenodo database (https://doi.org/10.5281/zenodo.6512224).

### 5.2 Computational methods

#### 5.2.1 Candidate selection

We selected a total of 21 putative *de novo* protein candidates. Ten are uncharacterized putative *de novo* proteins from *Homo sapiens* ^22^ and are referred to here as *HS*1‐10. Ten proteins originate from *Drosophila melanogaster* ^21^ and are referred to as *DM*1‐10. One is the functionally characterized putative de novo protein Atlas from *D. melanogaster*.^44^ The 21 candidates contain different levels of disorder and secondary structure elements (α‐helix, β‐sheet, and mixture of both) and different sequence lengths (see Figure 2). We selected only candidate sequences without exon/intron structure and without long single amino acid repeats. All putative *de novo* proteins have confirmed expression in their native organism.

#### 5.2.2 Predictions

We performed disorder predictions with IUPred2a^45^, ^46^ using default options *long disorder* for entire proteins. We calculated the average disorder score of the whole sequence and percentage of residues predicted to be disordered. The percentage of disorder was calculated by taking the amount of disordered residues (disorder score > 0.5) and dividing it by the sequence length of the protein. We also predicted average disorder and percentage of disordered residues with a disorder threshold of 0.8 (Figure S1). A python script was used to automate predictions and disorder proportion for all candidates. We performed α‐helix and β‐sheet predictions to verify the amount of disordered residues predicted by IUPred2a. Secondary structure predictions were performed with Porter 5.0 (SS3).^47^, ^48^ The predicted secondary structure elements for each residue were counted with a Javascript and divided by the total number of residues to obtain a percentage score for each structural element. DnaK binding sites were predicted using the ChaperISM suite (v1) in quantitative mode with default settings.^70^

### 5.3 Experimental methods

#### 5.3.1 Cloning of putative *de novo* candidates

Putative *de novo* candidates were synthesized as strings DNA from Twist Bioscience, San Francisco, codon optimzed for *E. coli* and without restriction sites used for cloning (BamHI, HindIII, NcoI, XhoI) inside the sequence. The wild‐type DNA for Atlas was provided by Geoff Findlay. To introduce restriction sites at the ends, we used different primers (a fasta file containing the DNA sequences and primer used can be found online on Zenodo (https://doi.org/10.5281/zenodo.6512224) For cloning into pHAT2 vector (N‐terminal 6xHis) we used restriction enzymes combination of BamHI/XhoI + HindIII, for pETM‐30 (N‐terminal 6xHis‐GST‐TEV), we used NcoI+HindIII. Both vectors were from the EMBL vector database, Heidelberg, introduced stop‐codon was TAA for all constructs. We digested the PCR product with both restriction enzymes respectively (FastDigest, Thermo Scientific) for 3 h at 37°C. Digest of the vector (1 h, 37°C) was purified from agarose gel (Zymo Research). We ligated both with an insert:vector ratio of 1:4 using Ligase (Thermo Scientific; 1 h, 22°C). The ligation mix was purified (Zymo Research) and 2 μl of the purified reaction mix was used to transform into 50 μl of chemically competent *E. coli* TOP10 cells. Cells were incubated for 30 min on ice, followed by a 90 sec heat‐shock at 42°C. 500 μl of LB‐Media (5 g yeast extract, 6 g tryptone, 5 g NaCl) was added for recovery and incubated for 1 h at 37°C. After incubation, the resuspended cell pellets were plated on LB‐agar containing 50 μg/ml ampicillin (AMP, Carl Roth, pHAT2, and EMBL vector database) or Kanamycin (KAN, Carl Roth, pETM‐30, and EMBL vector database) and incubated at 37°C over night.

Successful transformation was verified by colony PCR and sequencing at Microsynth, Seqlab, Germany. The plasmid DNA bearing the chaperone combinations GroEL/ES (pGro7) or DnaK/J/GrpE (pKJE) from Takara Biotech chaperone kit^71^, ^72^ were first transformed into *E. coli* Top10 cells and then into expression strains (BL21 Star™ (DE3) and T7 Express). Chaperone plasmid bearing cells were made chemically competent (Inoue method)^73^, ^74^ and used for transformation with the plasmid containing the target protein sequence. Final expression cells contained two plasmids: chaperone plasmid and target protein plasmid. The chaperone plasmids are chloramphenicol (CAM) resistant, so the double plasmid cells are either AMP + CAM (pHAT2, N‐terminal 6xHistag) or KAN + CAM (pETM‐30, N‐terminal GST‐tag) resistant.

#### 5.3.2 Test‐Expression of candidate *de novo* proteins

To identify in which strain and plasmid proteins were expressed we performed test expressions. 10 ml of LB + AMP + CAM or LB + KAN + CAM were inoculated from a glycerol stock of all three expression cells bearing both plasmids (target protein and chaperone) and grown until turbid (6–8 h, 37°C). We split the solutions into 3 × 3 ml and incubated for 30 min at different temperatures (37°C, 28°C, and 20°C) before adding IPTG (Carl Roth) for a final concentration of 0.5 mM and shaking over night. When using the cells with chaperone plasmids we made the following adjustment: L‐arabinose (final concentration 3 mM, Carl Roth) was added from the beginning for immediate induction of chaperone expression. Therefore, after inducing the de novo protein expression with IPTG the chaperones were already present in order to help folding the *de novo* proteins.

A total of 500 μL of each cell culture were centrifuged (15,000 rpm, 2 min). Pellets were resuspended and lyzed in 50 μl of a mix of Bugbuster and Lysonase (both Merck AG) through vortexing for 10 min. After centrifugation the supernatant was mixed with the same volume of SDS‐loading buffer (standard). The pellet was resuspended in 5x diluted Bugbuster, centrifuged, and resuspended in 50 μl SDS‐loading buffer. 15 μl of each fraction was loaded on an SDS‐PAGE, either 10% Bis‐Tris or 12.5% TGS, run on 200 V for 50 min and dyed using ReadyBlue™ staining.

For the final Western blots, the determined optimal combination of strain, expression vector, and chaperone plasmid were used. 20 ml cultures of 2YT + AMP + CAM or 2YT + KAN + CAM were inoculated with 1 ml of the overnight culture. L‐arabinose (final concentration 3 mM) was added to the samples, but not to the control without chaperones and grown at 37°C, 180 rpm for 4–6 hr until turbid. The cultures were incubated at 28°C, 180 rpm for 30 min before induction with IPTG (final concentration 0.5 mM) and incubated overnight under these conditions. Final samples were harvested and handled as prior performed test expressions.

#### 5.3.3 Western blot

The SDS‐PAGEs were run as described above but without ReadyBlue™staining. The gel was equilibrated in transfer buffer (20% Methanol) for a few seconds. A polyvinylidene fluoride (PVDF) membrane with a pore size of 0.22 μm was activated by methanol (2 min) and equilibrated in transfer buffer. The semi‐dry transfer was performed at 25 V for 30 min using the BioRad standard protocol. The membrane was blocked at room temperature for 1 hr using 5% bovine serum albumin BSA in phosphate‐buffered saline with tween (PBS‐T) then washed in PBS‐T and incubated for 1 h with anti‐His antibody (MA1‐21315‐HRP) diluted 1:500. For chemiluminescence, 0.5 ml luminol was mixed with 0.5 ml peroxide and distributed evenly on the membrane. Intensities of the different bands were measured in ImageJ after default background subtraction. The different fractions (soluble/insoluble of base, GroEL and DnaK) are calculated as fractions of the intensities of all relevant protein bands to also compare the amount of expression between the different conditions.

#### 5.3.4 Mass spectrometry

Tryptic digest followed by mass spectrometry for peptide detection of the candidate proteins was performed by the Core Unit Proteomics group of Prof. Dr. Simone König, UKM Muenster.

## AUTHOR CONTRIBUTIONS

**Lars A. Eicholt:** Data curation (lead); investigation (lead); validation (lead); visualization (lead); writing – original draft (lead). **Margaux Aubel:** Data curation (equal); investigation (equal); validation (equal); visualization (equal); writing – original draft (equal). **Katrin Berk:** Data curation (supporting); validation (supporting); writing – original draft (supporting). **Erich Bornberg‐Bauer:** Conceptualization (supporting); funding acquisition (lead); project administration (supporting); resources (lead); supervision (supporting); writing – original draft (supporting). **Andreas Lange:** Conceptualization (lead); investigation (supporting); project administration (lead); supervision (lead); validation (supporting); writing – original draft (supporting).

## CONFLICT OF INTERESTS

The authors declare no competing interests.

## Supporting information


**Figure S1** Percentage of average disorder as calculated with IUPred2a. All candidate *de novo* proteins used for expression experiments ordered by their average disorder level from left to right.

**Figure S2** Number of DnaK binding Heptamers as predicted with ChaperISM suite (v1) per candidate *de novo* protein. *DM*3 was expressed solubly with DnaK, but no binding sites were predicted.

**Figure S3** SDS‐PAGEs with all *D. melanogaser de novo* protein candidates co‐expressed with GroEL system and without. The red boxes indicate GroEL chaperone, green boxes the target protein.

**Figure S4** SDS‐PAGEs with solubly expressed *de novo* protein candidates of *H. sapiens* co‐expressed with GroEL system and without. The red boxes indicate GroEL chaperone, green boxes the target protein.

**Table S1** Names of the putative *de novo* proteins, annotated gene names (only for *D. melanogaster*) and genomic locations on genome assembly BDGP6.32 (*D. melanogaster*) and GRCh38.p13 (*H. sapiens*).
