# Supplementary figures and images for: Heterologous expression of naturally evolved putative de novo proteins with chaperones
Source: Protein Sci. 2022 Jul 13;31(8):e4371. doi: 10.1002/pro.4371 (PMC9278007; doi:10.1002/pro.4371)

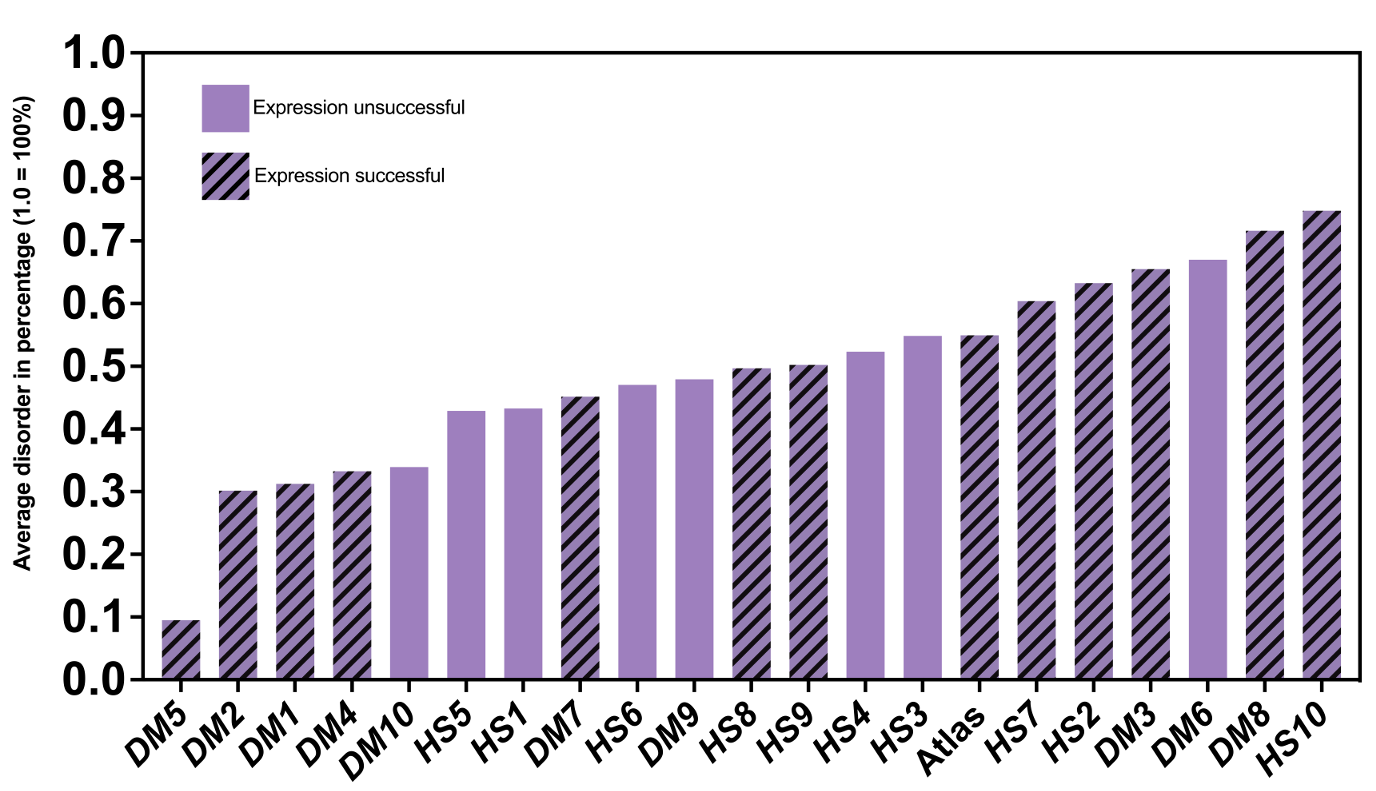

Supplement: Supplementary file 1 — Figure S1 Percentage of average disorder as calculated with IUPred2a. All candidate de novo proteins used for expression experiments ordered by their average disorder level from left to right. [file PRO-31-e4371-s004.tiff]

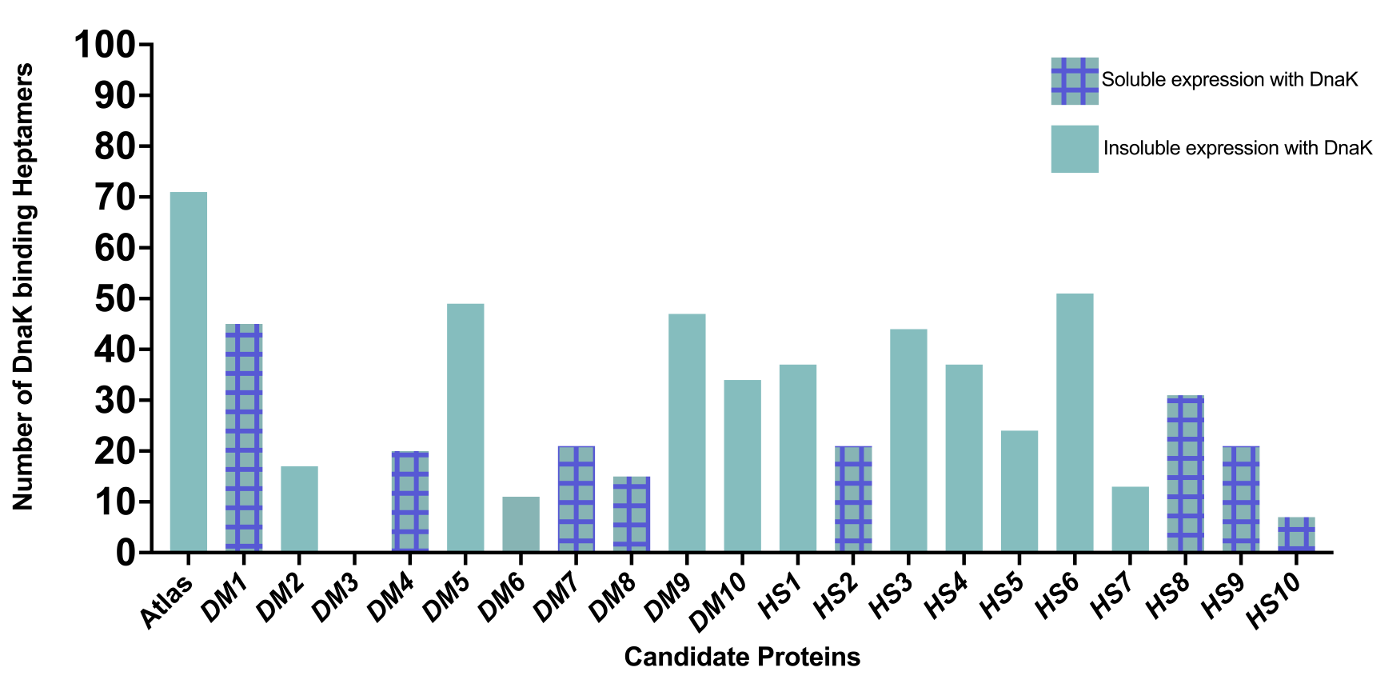

Supplement: Supplementary file 2 — Figure S2 Number of DnaK binding Heptamers as predicted with ChaperISM suite (v1) per candidate de novo protein. DM3 was expressed solubly with DnaK, but no binding sites were predicted. [file PRO-31-e4371-s002.tiff]

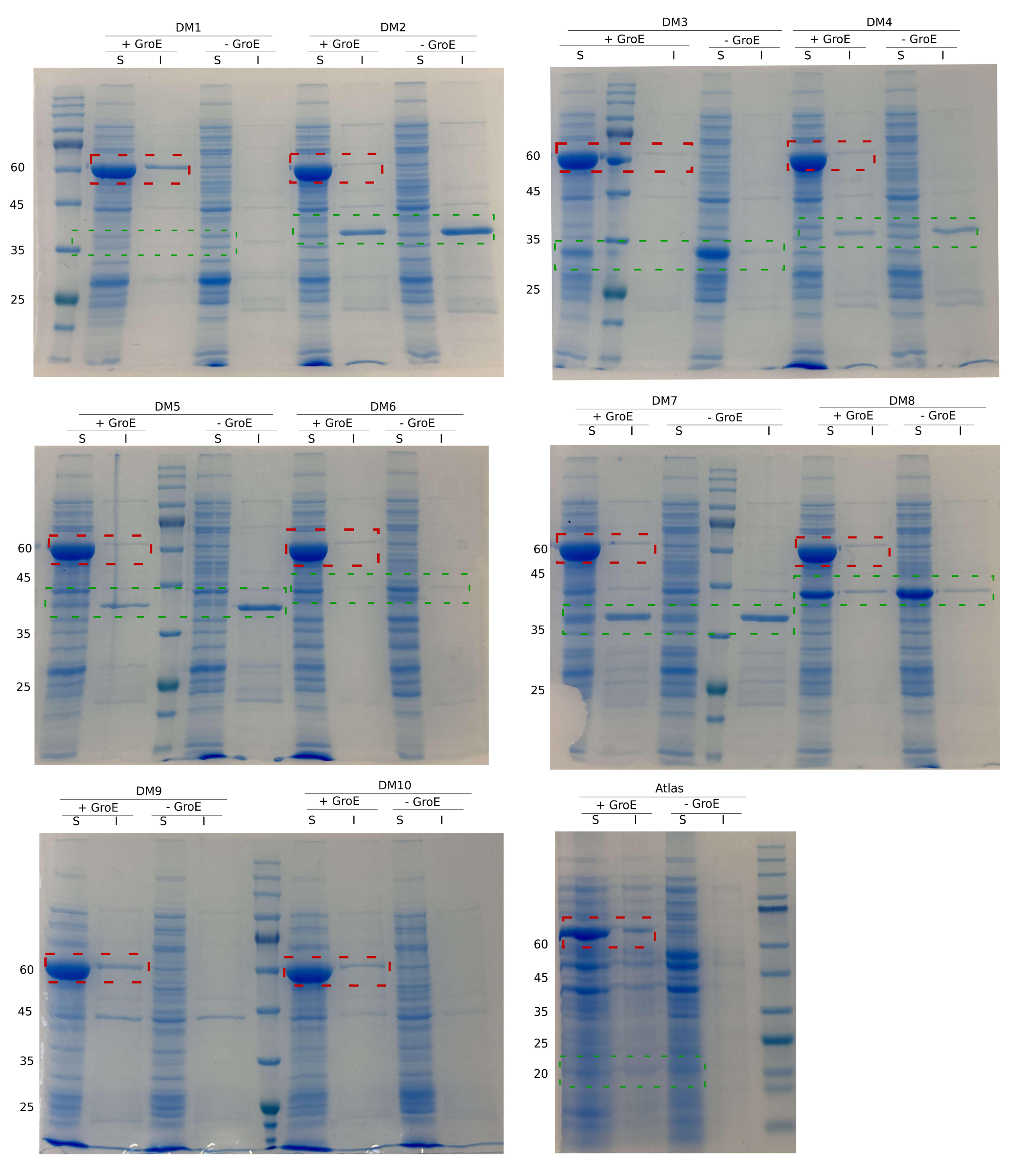

Supplement: Supplementary file 3 — Figure S3 SDS‐PAGEs with all D. melanogaser de novo protein candidates co‐expressed with GroEL system and without. The red boxes indicate GroEL chaperone, green boxes the target protein. [file PRO-31-e4371-s005.tiff]

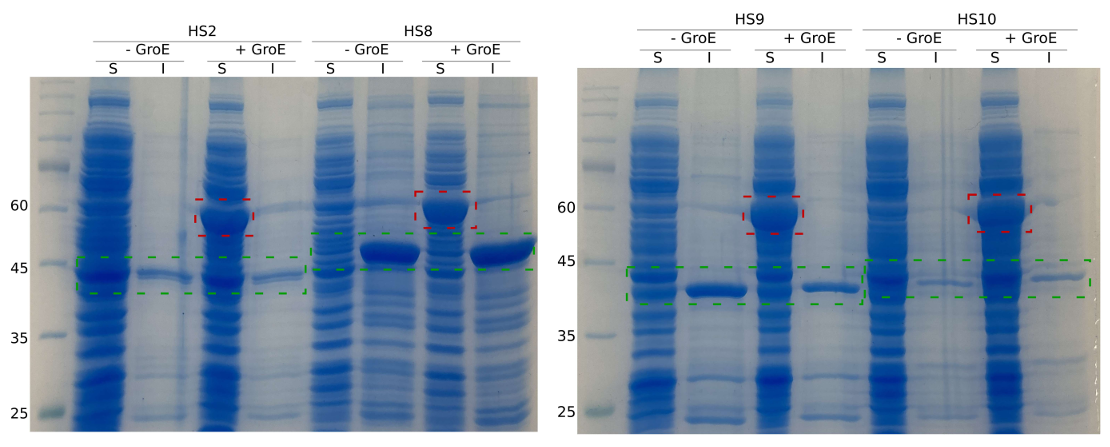

Supplement: Supplementary file 4 — Figure S4 SDS‐PAGEs with solubly expressed de novo protein candidates of H. sapiens co‐expressed with GroEL system and without. The red boxes indicate GroEL chaperone, green boxes the target protein. [file PRO-31-e4371-s003.tiff]
