# Supplementary material for: Heterologous expression of naturally evolved putative de novo proteins with chaperones
Source: Protein Sci. 2022 Jul 13;31(8):e4371. doi: 10.1002/pro.4371 (PMC9278007; doi:10.1002/pro.4371)
Supplement: Supplementary file 5 — Table S1 Names of the putative de novo proteins, annotated gene names (only for D. melanogaster) and genomic locations on genome assembly BDGP6.32 (D. melanogaster) and GRCh38.p13 (H. sapiens). [file PRO-31-e4371-s001.docx]

Table S1 Names of the putative *de novo* proteins, annotated gene names (only for *D. melanogaster*) and genomic locations on genome assembly BDGP6.32 (*D. melanogaster*) and GRCh38.p13 (*H. sapiens*).

| Name | Gene | Chromosome | Start | End | Origin |
| --- | --- | --- | --- | --- | --- |
| Atlas | CG13541 | 2R | 23284227 | 23285821 | *D. melanogaster* |
| *DM*1 | CG44259 | 3R | 17253581 | 17254129 | *D. melanogaster* |
| *DM*2 | CG44426 | X | 20978286 | 20978868 | *D. melanogaster* |
| *DM*3 | CG45691 | 2L | 17320250 | 17320712 | *D. melanogaster* |
| *DM*4 | CG45676 | 3R | 11418885 | 11419295 | *D. melanogaster* |
| *DM*5 | CG44815 | X | 9445815 | 9446212 | *D. melanogaster* |
| *DM*6 | CG42544 | 3R | 7304592 | 7304954 | *D. melanogaster* |
| *DM*7 | CG44569 | 2R | 19842495 | 19843668 | *D. melanogaster* |
| *DM*8 | CG44261 | 3R | 9407694 | 9408169 | *D. melanogaster* |
| *DM*9 | CG45095 | 3R | 25102553 | 25103617 | *D. melanogaster* |
| *DM*10 | CG43800 | 2L | 7232165 | 7232813 | *D. melanogaster* |
| *HS*1 |  | 13 | 114280607 | 114281009 | *H. sapiens* |
| *HS*2 |  | 11 | 47587158 | 47587581 | *H. sapiens* |
| *HS*3 |  | 10 | 63630509 | 63631028 | *H. sapiens* |
| *HS*4 |  | 9 | 70836395 | 70836767 | *H. sapiens* |
| *HS*5 |  | 7 | 44560904 | 44561255 | *H. sapiens* |
| *HS*6 |  | 3 | 46846292 | 46846856 | *H. sapiens* |
| *HS*7 |  | 17 | 82729322 | 82729919 | *H. sapiens* |
| *HS*8 |  | 8 | 1814237 | 1814609 | *H. sapiens* |
| *HS*9 |  | 16 | 2647236 | 2647602 | *H. sapiens* |
| *HS*10 |  | 19 | 10404723 | 10405134 | *H. sapiens* |
